# Secondary analysis of a randomized trial testing community health educator interventions for diabetes prevention among refugees with depression: effects on nutrition, physical activity and sleep

**DOI:** 10.1186/s12966-023-01509-y

**Published:** 2023-09-12

**Authors:** Julie A. Wagner, Angela Bermúdez-Millán, Thomas E. Buckley, Orfeu M. Buxton, Richard S. Feinn, Sengly Kong, Theanvy Kuoch, Lindsay Master, Mary F. Scully

**Affiliations:** 1 UConn Health, 263 Farmington Ave., Farmington, CT 06030 USA; 2 University of Connecticut School of Medicine, Farmington, CT USA; 3 University of Connecticut School of Pharmacy, Storrs, CT USA; 4 Pennsylvania State University, University Park, PA USA; 5 Quinnipiac University, Hamden, CT USA; 6 Khmer Health Advocates, West Hartford, CT USA

**Keywords:** Nutrition, Physical activity, Sleep, Depression, Refugee

## Abstract

**Background:**

Refugees have high levels of psychological distress that hamper lifestyle change efforts. We previously reported that community health educator (CHE) diabetes prevention interventions decreased HbA1c and depressive symptoms among Cambodian-American refugees with depression; this paper reports health behavior outcomes of those interventions.

**Methods:**

Participants were aged 35–75, Khmer speaking, at risk for diabetes, and met study criteria for likely depression by either a) antidepressant medication and/or b) prolonged elevated depressive symptoms. Participants were randomized to one of three CHE interventions: 1) lifestyle intervention called *Eat, Walk, Sleep* (EWS), 2) EWS plus medication therapy management with a pharmacist/CHE team (EWS + MTM), or, 3) social services (SS; control). Physical activity and sleep were measured with 7 days of actigraphy. Nutrition was measured as carbohydrates as reported in a culturally tailored food frequency questionnaire. Assessments were at baseline, end point (12 months), and follow-up (15 months).

**Results:**

The *n* = 188 participants were 78% female, average age of 55 years, half had a household income < $20,000, and modal education was 7.0 years. Individuals in the two treatment groups that received the EWS intervention significantly increased their brown rice consumption (*p* < .001, Cohen’s d = 0.76) and their moderate-to-vigorous activity (*p* = .039, d = 0.32). No intervention changed sleep duration, timing, efficiency or wake after sleep onset. Across groups, individuals who increased brown rice consumption, increased vigorous activity and decreased total sleep time variability showed decreased HbA1c, with small effect sizes.

**Conclusions:**

CHEs may improve nutrition and physical activity in refugees with depression but more intensive interventions may be required to impact sleep. Improvements in all three behaviors appear to be associated with HbA1c lowering

**Trial registration:**

ClinicalTrials.gov identifier NCT02502929.

## Introduction

Unhealthy lifestyle is a strong risk factor for type 2 diabetes. For example, higher total dietary intake of carbohydrate [1], low physical activity [2] and sleeping < 6 h per night each increase risk for diabetes. There is also evidence for the corollary, i.e., that better diet quality, higher physical activity, and adequate sleep [3] can mitigate genetic risk for diabetes.

Each of these health behaviors is made more difficult in the setting of depression. For example, high sugar intake, difficulty increasing physical activity, and sleep disruption are all associated with depressive symptoms [4–7]. The Diabetes Prevention Program (DPP) showed that lifestyle intervention was effective for diabetes delay and prevention [8]. Yet, data are lacking from rigorous randomized trials showing the ability of lifestyle change interventions to improve nutrition, physical activity, and sleep in people with depression.

Such data are particularly important for refugee groups due to their high rates of both depression and diabetes. Refugees are people who have fled war, violence, conflict or persecution and have crossed an international border to find safety in another country. The first Cambodian refugees were resettled in the US approximately 40 years ago fleeing the genocidal Pol Pot regime and their rates of past-year major depressive disorder are 51% [9]. For many Cambodian Americans the depression becomes chronic, with pre-migration trauma increasing odds of major depressive disorder even decades after resettlement [9]. As they age, the disproportionate burden of diabetes becomes evident. Age- and sex-adjusted rates of type 2 diabetes for Cambodian Americans are more than double the US national average [10, 11].

With their involuntary migration, many refugees experience westernization and urbanization in an industrialized host country along with respective changes in eating, physical activity, and sleep patterns. Diabetes appears higher in migrant populations than in their host countries [12]. Improving health behaviors and decreasing risk for diabetes is challenged by burden of migration, emotional distress, social determinants of health, and cultural and linguistic differences [13, 14]. Whereas the population of Cambodian Americans specifically is relatively small, involuntary migration due to armed conflict and economic privation is a common and global problem. In 2020, there were an estimated 281 million international migrants, equating to 3.6% of the global population [15]. Lessons learned from the Cambodian American experience may be informative to other refugee and migrant groups [16].

*Eat, Walk, Sleep* (EWS) is a cardiometabolic lifestyle curriculum that was created by and for Cambodian Americans and was designed to be delivered by lay health workers [17, 18]. EWS emphasizes trauma-informed changes to nutrition, physical activity, and sleep habits. Medication therapy management (MTM) is a pharmacist intervention aimed at resolving drug therapy problems that we have adapted to be delivered by cross-cultural teams of pharmacists paired with community health workers [19, 20]. In a sample of Cambodian Americans with depression and high risk for developing diabetes, we compared the efficacy of EWS vs. EWS plus medication therapy management (EWS + MTM) vs social services (SS, control condition). Study assessments were at baseline, 12-months (post-treatment) and 15-months (follow-up).

In a previous report of our primary outcomes, we showed that, relative to social services (SS), EWS + MTM improved HbA1c while both EWS and EWS + MTM decreased depression [21]. Below we report planned analyses of our secondary outcomes, i.e., key lifestyle behaviors including 1) daily grams of carbohydrate from rice, 2) physical activity and sedentary behavior per actigraphy, and, 3) sleep per actigraphy. We hypothesized that both groups that received the behavior change intervention (EWS and EWS + MTM) would show more improvement in nutrition, physical activity, and sleep than participants who did not (SS). In an exploratory fashion, we also examined which behavior changes were associated with improved primary outcomes HbA1c, depressive symptoms, and insulin resistance.

## Methods

Diabetes Risk Reduction through Eat, Walk, Sleep and Medication Therapy Management (DREAM) was a randomized, controlled trial to compare the efficacy of EWS vs. EWS + MTM vs social services (SS, control condition). Assessments were at baseline, endpoint (12-months) and follow-up (15-months). The study was pre-registered at ClinicalTrials.gov identifier: NCT02502929.

Participants randomized to SS were assessed for social service needs such as food and housing assistance and then provided support for any unmet needs over 12 months as needed. Participants assigned to EWS or EWS + MTM received their interventions over the course of 12-months. A post-intervention endpoint assessment was conducted at 12-months. A ‘booster’ EWS session (and MTM session for those assigned to EWS + MTM) occurred between 12- and 15-months. A follow-up assessment was conducted 15 months after baseline.

All staff who had direct contact with participants were born in Cambodia, bilingual and bicultural. To minimize bias, lay health workers were divided into two roles. To minimize bias, community health *workers* (CHWs) conducted all data collection while community health *educators* (CHEs) delivered all intervention sessions. Both CHWs and CHEs conducted recruitment, screening and consenting.

The study was conducted according to the World Medical Association Declaration of Helsinki and approved by the UConn Health institutional review board. Participants signed written informed consent forms in their preferred language (Khmer or English) and provided written HIPAA authorization and a release of information for study staff contact with their healthcare provider.

### Participants

DREAM recruited through referrals from clinicians and social service agencies; outreach at large cultural gatherings such as Cambodian new year celebrations; posters at local Khmer businesses; holding special events at relevant temples and churches. Inclusion criteria were: 1) aged 35–75; 2) Cambodian or Cambodian-American; 3) Khmer speaking; 4) currently living in Connecticut, Massachusetts, or Rhode Island (northeastern U.S.); 5) lived in Cambodia during the Pol Pot regime (1975–1979); 6) ambulatory; 7) consumed meals by mouth; 8) elevated risk for diabetes according to a modified version of the American Diabetes Association Risk Test [22–24]. Note that the sample was selected for elevated diabetes risk, not for pre-diabetes per se. Participants were also required to meet criteria for depression by a) current antidepressant medication, and/or, b) elevated depressive symptoms indicative of likely major depressive disorder on the Khmer language Hopkins Symptom Checklist [25] with elevated symptoms on two occasions that were two weeks apart during a study screening and eligibility period. Exclusion criteria were: type 2 diabetes; seeing or hearing problems that would interfere with group sessions; major medical problems requiring intensive treatment; pregnancy or planning pregnancy; serious thinking or memory problems (e.g., schizophrenia or dementia); and 3 or more days in a psychiatric hospital or self-harm in the past 2 years. Sample size was determined using a power calculation to detect an effect on the primary outcomes at endpoint (12 months) as previously described [21]. Recruitment began in March 2016 and data collection ended in September 2020.

### Procedures

CHW data collectors conducted assessments in a private setting at a location of the participant’s choice, either in-home or at a clinic or social service agency. The CHWs administered surveys verbally and recorded responses in Remote Electronic Data Capture (REDCap) [26] using a tablet. Participants were paid $10 USD in gift cards to a local pharmacy for completing the surveys. CHWs obtained anthropometrics and blood pressures according to standard practice as previously described [21, 27]. Participants were asked to wear accelerometers on their non-dominant wrist (for sleep measurement) and hip (for physical activity measurement) for seven consecutive days. CHWs instructed the participants to wear the devices at all times, day and night, except when the devices may get wet. On the seventh day, CHWs met with the participant for the return and subsequent download of devices. Next, CHWs removed instruments from participants and compensated them $10 for actigraphy. CHWs then returned the devices to the laboratory for data retrieval. On a separate day, participants presented to a nearby Quest Diagnostics to provide a fasting blood sample and were compensated an additional $10.

### Randomization

After all components of the baseline assessments were complete (i.e., surveys, anthropometrics and blood pressure, actigraphy, bloodwork), participants were individually randomized using an urn randomization [28] computer program that balanced the three treatment arms on gender, age, symptoms of post-traumatic stress disorder, and site (Connecticut, Rhode Island, Massachusetts) in a 2:2:1 ratio (EWS:EWS + MTM:SS) as previously described [29].

### Interventions

Participants who were randomized to EWS or EWS + MTM were assigned to receive 3 individual EWS sessions and 24 group EWS sessions delivered by CHEs. The three individual sessions cover behavioral targets included eating no more than 1 small bowl of (brown) rice per meal, walking at least 30 min per day on 6 days per week, and getting 7–9 h of restful sleep per night. The curriculum addresses the influence of genocide and resilience on health behaviors. After completing individual EWS sessions, participants started 1-h EWS group sessions and were asked to attend over 12 months. Each began with group discussion of traditional Khmer concepts of health. Sessions were conversational and activity based, including relaxation, supervised exercise, and cooking components. Each session included SMART (specific, measurable, achievable, realistic, timely) goal setting. Sessions were held in community settings convenient to the participants (e.g., social service agencies, temples). A full description of interventionists, intervention sessions, and settings has been previously reported [29].

Participants assigned to EWS + MTM attended group EWS sessions with participants in the EWS arm. In addition, they received at least 3 MTM sessions between baseline and post assessment. A booster MTM session was also scheduled between endpoint (12-month) and follow-up (15-month) assessments. MTM followed guidelines of the American Pharmacist Association [30] and the pharmacists delivering MTM were either certified in MTM or relevant specialties. Patients and CHEs were together face-to-face and communicated with the pharmacist via telemedicine using secure, high-definition videoconferencing. Thereafter, the three met as clinically indicated, but at least two more times over the course of the 12-month intervention, and once again for a ‘booster’ session between 12- and 15-months. The pharmacist relayed relevant clinical information to the prescriber. For participants whose labwork indicated prediabetes (i.e., HbA1c 5.7%-6.4% and/or fasting glucose 100-125 mg/dL), the pharmacist sent the prescriber recommendations to repeat the test and consider initiating Metformin.

Participants who were assigned to SS were assessed for any social service needs including food or housing assistance, referral to a healthcare provider, tax preparation, or citizenship applications and CHEs/CHWs followed up to address the need. For equity, after they completed their follow-up assessment, participants randomized to SS received a single, abbreviated EWS session and results of their lab work, along with results to their healthcare provider.

### Measures

#### EAT behaviors

We assessed three domains of nutrition behaviors important for cardiometabolic disease. Using community-based, participatory methods we developed a brief food frequency questionnaire that was tailored for the Cambodian American diet. It focused on the most commonly consumed sources of carbohydrate (rice, rice products such as rice porridge, sweetened condensed milk, fruit juices, fruit drinks, fruit shakes (e.g., taro bubble tea), and regular soda). Frequency was assessed using a timeframe of the previous 3 months. Amounts were estimated by participants using empty culturally appropriate bowls, plates, teaspoons, tablespoons and drinking glasses that were standardized for volume. Using a validated computerized food database “ESHA—the Food Processor Nutrition Analysis program” [31], responses were converted to daily totals, i.e., carbohydrates from white rice, brown rice and sweet drinks in grams/day.

#### WALK behaviors

Physical activity and sedentary time were measured objectively with actigraphy. Participants wore tri-axial accelerometer (Actigraph GT3X, Actigraph, Pensacola, FL) on the hip using an elastic belt or clip. Data collected at 80 Hz were downloaded (Actilife software, v6.13.3, Actigraph, Pensacola, FL, USA). Activity counts were analyzed at the minute-level from the vertical axis of the device. Classification of activity was determined using cut-points to categorize minutes in sedentary behavior (< 100 counts per minute), light (100–2019 CPM), moderate (2020–5998 CPM), and vigorous physical activity (5999 + CPM) [32, 33]. Nonwear was defined by an interval of at least 60 consecutive minutes of zero activity intensity counts, with allowance for 1–2 min of counts between 0 and 100 [32, 33].

Physical activity actigraphy data were merged with sleep actigraphy data at the minute-level to determine “daytime” physical activity measures between morning sleep offset and nighttime sleep onset, which identifies true sedentary behavior that does not include nighttime sleep or napping. Physical activity days were determined invalid if there were > 25% non-wear minutes during the “daytime” portion of the day, or if there was < 20 h within the day that was “forced-ended” due to an invalid sleep period. Sleep and physical activity actigraphy temporal alignment and valid day criteria methods are detailed elsewhere [34].

#### SLEEP behaviors

Sleep was measured objectively with actigraphy and subjectively with self-report. Objective sleep measures were collected using an accelerometer (Actiwatch Spectrum Plus; Philips-Respironics, Murrysville, PA) worn on the non-dominant wrist for one week. Data were collected and exported at 30-s epochs, where at least two independent scorers visually determined validity of data and set sleep intervals for periods greater or equal to 20 min using a graphical user interface. Together, the scorers reviewed each recording to determine the final number of valid days and sleep intervals using a validated scoring procedure [34, 35]. Using data from the device’s “on-wrist” sensor, a sleep actigraphy day was determined invalid and no sleep interval was set if there were ≥ 4 total hours of off-wrist time, with the exception of the first and last day (device should be worn at least 2 h before sleep onset on the first day). Outcomes included mean nighttime total sleep time (TST), sleep maintenance efficiency (the percentage of time spent asleep after first falling asleep [sleep onset] and before waking up [sleep offset] the next day), mean nighttime wake after sleep onset (WASO), standard deviation of 24-h TST, and standard deviation of sleep timing (clock-hour midpoint for nighttime sleep).

### Data analysis

We were interested in whether the EWS intervention improved key eat, walk, and sleep behaviors. In pre-planned analyses approved by the funder, we compared changes in key eat, walk, and sleep behaviors between the 2 groups that received EWS and the group that did not, across timepoints. Independent variables were experimental assignment (SS vs EWS vs EWS + MTM) and timepoint (baseline, 12-months, 15-months) and the dependent variables were as above. Descriptive statistics include means with standard deviations. Linear mixed models was used to test for change between groups. We first tested for differences between the two groups that received EWS intervention, i.e., EWS and EWS + MTM groups and, as expected, they showed a similar profile on the outcome variables. Thus, they were combined in the testing using contrasts statement and compared with the SS group. The linear contrasts tested for differences between the combined EWS/EWS + MTM group versus the SS group in the change from baseline to both the 12-month and 15-month time points. The physical activity and nutrition outcomes deviated severely from normality and typical transformations were unsuccessful in rectifying the issue. Therefore, values were converted to ranks and the mixed models were applied to the ranked data. For effect size Cohen’s d is reported for differences between experimental arms (EWS/EWS + MTM) and control arm (SS) at 12-months and 15-months.

The nonparametric Spearman correlation was used to explore the relationship between change from baseline to 12-months in sleep, physical activity, and nutrition with change in primary outcomes of HbA1c, depressive symptoms, and logHOMA-IR. A significant correlation would suggest that the respective behavior may be mechanistically related to the primary outcomes, thus supporting targeting the behavior. In the EWS + MTM group, we calculated the proportion of participants for whom Metformin was recommended, who actually initiated Metformin treatment at any point across the 15 months. All available observations were used in the analyses and missing imputation was not performed as study retention was excellent (96% at 15-month follow-up). Statistical significance was set at an alpha level of 0.05. Analyses were conducted in SPSS v28 and syntax code is stored on the OSF link https://osf.io/9g73k/.

## Results

Of 540 individuals screened, 206 recruits were consented and 188 participants were randomized. The CONSORT diagram and details of baseline characteristics have been reported elsewhere [29]. At baseline there were no significant differences between groups in demographic or clinical characteristics. As previously reported [29] participants averaged 55 years old, had resided in the US for an average of 28 years, 78% was female, approximately 1/2 had a household income below $20,000, and the average years of formal education was 7.0 years. Most (60%) had public insurance and the average BMI was 27 with 83% meeting the threshold of overweight or obese. Over half (54%) had elevated depressive symptoms and about one-third were taking antidepressant medication. We have previously reported excellent retention, attendance, and treatment fidelity (see [29]).

Sixteen participants in the EWS + MTM arm had baseline HbA1c and/or fasting glucose that exceeded the cutoff for prediabetes. The healthcare providers of all of them received recommendations to repeat the labwork and/or initiate metformin, depending on the clinical judgment of the pharmacist. Assessments at endpoint (12-months) and follow-up (15-months) indicate that none of these participants initiated metformin over the course of the study.

Descriptive statistics for the nutrition outcomes are shown in Table 1. All outcomes, except brown rice, had a significant conditional main effect for time point, where all three groups demonstrated improvement. For brown rice the change in consumption differed between the SS and intervention arms from baseline to 12-month (*p* < 0.001) and also at 15-months (*p* < 0.001). There was a substantial increase in consumption of brown rice for the EWS arms not seen in the SS group.

**Table 1:** Mean (± SD) nutrition outcomes of each group by timepoint in grams

**Nutrition**					
**Outcome**	Baseline	12-Month	15-Month	*P*-Values	Cohen’s d
**White Rice**					
Control	257.5 ± 211.8	193.8 ± 192.8	182.7 ± 165.1	12m = .139	0.23
EWS	274.4 ± 200.4	147.0 ± 150.1	146.0 ± 123.2	15m = .155	0.22
EWS + MTM	271.9 ± 195.6	142.3 ± 108.7	167.8 ± 156.7		
**Brown Rice**					
Control	32.9 ± 115.1	24.6 ± 82.2	13.6 ± 50.7	12m = < .001	0.76
EWS	16.5 ± 43.9	72.2 ± 92.6	75.3 ± 100.7	15m = < .001	0.74
EWS + MTM	29.9 ± 57.3	73.7 ± 87.2	72.0 ± 118.4		
**Sweet Drinks**					
Control	43.2 ± 62.3	27.1 ± 32.9	32.4 ± 44.1	12m = .958	0.01
EWS	32.1 ± 43.2	22.4 ± 30.9	23.6 ± 38.4	15m = .133	0.23
EWS + MTM	39.8 ± 86.3	32.3 ± 41.4	19.4 ± 30.1		
**Carbohydrates**					
Control	352.0 ± 313.5	252.3 ± 189.4	230.4 ± 166.6	12m = .506	0.10
EWS	326.3 ± 205.1	265.2 ± 199.3	271.0 ± 229.5	15m = .780	0.04
EWS + MTM	347.2 ± 212.4	265.8 ± 181.7	276.2 ± 246.7		

*EP* Endpoint (12 months, *FU* Follow-Up (15 months)

Descriptive statistics for physical activity actigraphy outcomes are shown in Table 2. The plurality of time was spent in sedentary activity and any physical activity was mostly light. At endpoint the two intervention arms increased their moderate activity more than SS (*p* = 0.052) and in combination with vigorous activity (moderate/vigorous physical activity or MVPA) the change from baseline to endpoint was statistically significant (MVPA *p* = 0.039).

**Table 2:** Mean (± SD) physical actigraphy outcomes of each group by timepoint in minutes

**Physical Activity**					
**Outcome**	Baseline	12-Month	15-Month	*P*-Values	Cohen’s d
**Sedentary Time**					
Control	490.6 ± 117.4	518.4 ± 119.3	526.5 ± 113.6	12m = .099	0.41
EWS	514.7 ± 100.2	495.5 ± 99.9	530.3 ± 99.8	15m = .970	0.01
EWS + MTM	533.4 ± 104.2	532.0 ± 98.2	556.8 ± 94.2		
**Light**					
Control	364.2 ± 108.3	363.3 ± 96.9	366.3 ± 118.4	12m = .130	0.24
EWS	391.5 ± 125.7	400.5 ± 105.7	383.7 ± 114.6	15m = .210	0.19
EWS + MTM	365.2 ± 100.5	378.0 ± 96.2	356.0 ± 88.9		
**Moderate**					
Control	27.5 ± 82.7	28.2 ± 82.1	22.2 ± 61.7	12m = .052	0.30
EWS	14.9 ± 15.2	19.6 ± 21.0	16.4 ± 15.1	15m = .310	0.16
EWS + MTM	14.6 ± 18.0	21.0 ± 21.2	14.1 ± 13.2		
**Vigorous**					
Control	1.69 ± 6.56	2.00 ± 10.2	0.41 ± 1.65	12m = .477	0.11
EWS	0.40 ± 2.21	0.34 ± 1.53	0.30 ± 1.13	15m = .274	0.17
EWS + MTM	0.08 ± 0.27	0.14 ± 0.49	0.12 ± 0.52		
**MVPA**					
Control	29.2 ± 87.9	30.2 ± 92.0	22.7 ± 62.6	12m = .039	0.32
EWS	15.3 ± 15.4	20.0 ± 49.3	16.7 ± 15.5	15m = .250	0.18
EWS + MTM	14.6 ± 18.1	21.1 ± 21.2	14.2 ± 13.3		

*EP* Endpoint (12 months, *FU* Follow-Up (15 months)

Table 3 shows the descriptive statistics for the sleep actigraphy outcomes. There were no statistically significant differences between SS and the intervention groups on change from baseline to endpoint or follow-up for any of the sleep outcomes. There were significant marginal main effects for time point on WASO (*p* < 0.001) and sleep efficiency (*p* < 0.001), such that both outcomes improved from baseline, but this was experienced by all groups.

**Table 3:** Mean (± SD) sleep actigraphy outcomes of each group by timepoint

**Sleep**					
**Outcome**	Baseline	12-Month	15-Month	*P*-Values	Cohen’s d
**Total Sleep Time in minutes**					
Control	469.8 ± 71.2	451.5 ± 63.5	450.9 ± 70.0	12m = .578	0.09
EWS	449.5 ± 83.3	436.7 ± 73.3	437.7 ± 70.8	15m = .314	0.16
EWS + MTM	450.0 ± 79.9	441.5 ± 63.9	437.8 ± 66.9		
**WASO in minutes**					
Control	59.0 ± 27.9	46.1 ± 18.2	47.9 ± 24.1	12m = .160	0.22
EWS	58.7 ± 28.8	50.7 ± 23.3	49.3 ± 21.9	15m = .199	0.20
EWS + MTM	52.6 ± 22.7	48.1 ± 21.3	47.2 ± 20.5		
**TST Variability in minutes**					
Control	72.5 ± 31.9	78.5 ± 37.1	70.9 ± 35.9	12m = .154	0.22
EWS	78.5 ± 35.3	72.7 ± 26.2	80.6 ± 43.4	15m = .701	0.06
EWS + MTM	86.7 ± 42.0	84.6 ± 33.2	86.1 ± 38.4		
**Midpoint Variability in hours**					
Control	0.78 ± 0.39	0.87 ± 0.55	0.92 ± 1.03	12m = .187	0.20
EWS	1.04 ± 1.36	1.06 ± 1.22	0.88 ± 0.64	15m = .085	0.27
EWS + MTM	1.03 ± 0.80	0.94 ± 0.48	0.98 ± 0.72		
**Sleep Efficiency %**					
Control	87.8 ± 5.1	89.9 ± 3.7	89.8 ± 4.0	12m = .157	0.22
EWS	87.7 ± 4.6	88.8 ± 4.2	89.0 ± 4.0	15m = .163	0.22
EWS + MTM	88.5 ± 3.9	89.3 ± 4.0	89.3 ± 4.0		

*EP* Endpoint (12 months, *FU* Follow-Up (15 months)

The Spearman correlations between the change from baseline to 12-month for the actigraphy and nutrition variables with the change from baseline to 12-month for HbA1c and depressive symptoms are shown in Table 4. Among the sleep actigraphy variables there was a significant correlation between total sleep time variability and HbA1c (*r* = 0.174, *p* = 0.026), such that decrease in variability was associated with a decrease in HbA1c. Among the physical activity variables change in vigorous activity was correlated with change in HbA1c (*r* = -0.208, *p* = 0.008), such that increase in physical activity was associated with lowering of HbA1c. Among the nutrition variables, both brown rice consumption (*r* = -0.262, *p* = 0.003) and total carbohydrates (*r* = -0.224, *p* = 0.007) were correlated with HbA1c. For both, increases from baseline to 12-month were associated with decrease in HbA1c.

**Table 4:** Spearman correlation between actigraphy & nutrition change scores with primary change scores

	**A1c**		**Depression**		**ln HOMA**	
	rho	*p*-value	rho	*p*-value	rho	*p*-value
**Sleep Actigraphy**						
Total Sleep Time	-.089	.212	.020	.799	-.004	.963
WASO	-.072	.359	.006	.934	.007	.929
TST Variability	.174	.026	.023	.765	.011	.889
Midpoint Variability	.129	.100	.038	.624	.137	.079
Sleep Efficiency	-.009	.908	-.006	.935	-.057	.465
**Physical Activity Actigraphy**						
Sedentary	.118	.136	-.071	.365	.036	.648
Light	-.032	.686	-.081	.302	-.102	.198
Moderate	.006	.937	-.006	.941	-.073	.362
Vigorous	-.208	.008	.137	.079	-.049	.540
MVPA	-.006	.937	-.003	.973	-.071	.369
**Nutrition**						
White Rice	-.098	.247	.058	.488	-.053	.535
Brown Rice	-.262	.003	-.005	.956	-.139	.116
Sweet Drinks	-.128	.131	-.039	.644	-.006	.943
Carbohydrates	-.224	.007	.042	.613	-.043	.608

Change score (Δ) is difference from baseline to 12-month

## Discussion

The main findings from this study are that individuals who received the EWS lifestyle intervention successfully increased their brown rice consumption and their moderate-to-vigorous activity. And whereas we previously reported that the intervention improved self-reported sleep quality, here we found that it did not impact objective sleep duration, timing, efficiency or WASO. Across groups, individuals who increased brown rice consumption, increased vigorous activity and decreased total sleep time variability showed improved HbA1c. This is the first randomized trial specifically designed to reduce risk for diabetes in refugees with depression [16]. Lifestyle change for refugees must consider the psychological experiences of hunger and starvation as they pertain to nutrition, forced labor as it pertains to physical activity, and trauma symptoms and nightmares as they pertain to sleep [36].

Healthy nutrition changes are complicated by historical experiences of nutritional hardship. Refugee households may experience nutritional “double burden”. The double burden of malnutrition is characterized by the coexistence of undernutrition along with overweight, obesity or diet-related non-communicable diseases, within individuals, households and populations, and across the life-course [37]. Double burden is not uncommon in populations that are dependent upon food assistance for survival. The quality of such diets may contribute to both nutritional extremes. The EWS curriculum acknowledges and even celebrates the centrality of rice in the Cambodian culture (the word for “eat” in Khmer is the same as “rice”). Therefore, rather than encouraging elimination of white rice, our curriculum emphasized the Buddhist concept of balance (not too much, not too little) and a return to ancestral consumption of brown, instead of white, rice. Our approach resulted in moderate-to-large sized effect in increased brown rice at 12- and 15-months. Brown rice is higher in fiber and micronutrients and provokes a less steep insulin response than white rice. Although brown rice was eaten throughout history in Cambodia, in modern times it has become associated with poverty and prison food.

Eating in refugee populations is also influenced by high levels of post-traumatic stress symptoms. Cambodians specifically endured famine and forced starvation at the hands of Khmer Rouge. Starvation is often described as one of the most traumatic aspects of the Pol Pot regime. Deliberate withholding of food and forced communal meals to regulate intake were widespread. Individuals with post-traumatic stress disorder (PTSD) exhibit more emotional eating in response to stressors than those without PTSD [38–40]. A recent study showed that Cambodian refugees in the USA who experienced extensive food deprivation or insecurity may be more likely to engage in unhealthful eating practices and to be overweight or obese than are those who experienced less extreme food deprivation or insecurity [39].

Physical activity is also complicated by a history of trauma. Individuals with symptoms of posttraumatic stress may tend to seek physiological quiescence and avoid physical arousal, including the increased heart rate, respiration, and perspiration caused by physical activity. Cambodians experienced forced labor under the Khmer Rouge, with long hours of inhumane work such as digging ditches and breaking rocks in intense heat. Thus, physical activity may trigger post-traumatic symptoms. We should note that EWS participants were not only willing, but enthusiastic, to join in group exercise as part of the EWS sessions. Actigraphy showed that the physical activity during this structured, supervised, and supportive setting successfully generalized to more moderate-to-vigorous activity in daily life outside of intervention sessions. The effect sizes for increases in physical activity at 12-months were small-to-moderate.

Individuals with mood disorders and trauma history often experience sleep disturbance [41]. For example, among traumatized Cambodians, common clinical complaints include nightmares, visitations by ghosts, sleep paralysis, and dreams of the dead [42–44]. We previously reported improved self-reported sleep quality among our intervention groups [45]. That we were not also able to improve objective sleep indicators suggests that more intensive cognitive behavioral treatments and/or medications may be indicated.

In exploratory analyses, we examined how changes in health behaviors were associated with changes that we have previously reported in our primary outcomes, i.e., HbA1c, depressive symptoms, and insulin resistance [21, 46]. Findings show that changes to all three health behaviors – nutrition, physical activity, and sleep – may promote lowering HbA1c. Specifically, increased brown rice, increased vigorous activity, and more regular sleep duration were all associated with lowering of HbA1c, each with a small effect size. The recommendation for adequate sleep in adults includes obtain 7 or more hours of sleep per night *on a regular basis*, for optimal health and well-being [47]. Our findings suggest that the ‘regular basis’ may be particularly important for HbA1c.

We were surprised that no health behavior changes were associated with improved depression or change in insulin resistance. We hypothesize that the improvements in depression experienced by the intervention groups, previously reported [21], were caused by other intervention factors such as behavioral activation and increased socialization. We were also surprised, and disappointed, that no participants in the EWS + MTM group initiated metformin, despite the fact that their providers received laboratory results and accompanying recommendations from study pharmacists. Metformin, an α-glucosidase inhibitor that reduces insulin resistance, has the longest history of safety data for diabetes prevention [48] and many healthcare providers follow American Diabetes Association guidelines which recommend metformin for the prevention of type 2 diabetes in adults at high risk [48]. Clinical inertia is a well-known problem in diabetes and prediabetes and it been documented that ethnic minority patients with multiple morbidities experience the longest delays in prescription of first line treatment [49].

## Limitations and conclusions

Because the randomized trial was powered to detect group changes in our primary outcomes—HbA1c, insulin resistance, and depressive symptoms—these secondary analyses may be limited by low statistical power. For the secondary outcomes reported here, the sample size had acceptable power (80%) to detect approximately a medium size effect (d ~ 0.5) and so was underpowered to detect small effects. Like all self-reports, our food frequency questionnaire may be subject to demand characteristics. The period of follow-up (3 months after post-treatment) may not adequately test the durability of effects. The details of the Cambodian American experience may not apply to all refugee groups, especially younger and newly arriving groups.

Modifying nutrition, physical activity and sleep is important for delaying or preventing a rise in HbA1c among high-risk individuals [50]. As mass migration from one region of the world to another continues due to famine, climate and armed conflict, lifestyle interventions based on EWS principles hold promise for mitigating diabetes risk in other refugee populations [51].

## Data Availability

The datasets used and/or analyzed during the current study are available from the corresponding author on reasonable request. Analyses were conducted in SPSS v28 and syntax code is stored on the OSF link https://osf.io/9g73k/. The primary materials used to deliver the intervention are presented in a public archive. Home | Diabetes Research (uconn.edu).

## Abbreviations

DPP: Diabetes prevention program
EWS: Eat, Walk, Sleep
MTM: Medication therapy management
SS: Social support
DREAM: Diabetes Risk Reduction through Eat, Walk, Sleep and Medication Therapy Management
CHW: Community health worker
CHE: Community health educator
REDCap: Remote electronic data capture
SMART: Specific, measurable, achievable, realistic, and timely
logHOMA-IR: Homeostasis model assessment of insulin resistance
MVPA: Moderate/vigorous physical activity
WASO: Wake after sleep onset
PTSD: Post-traumatic stress disorder
